# Modeling endodermal organ development and diseases using human pluripotent stem cell-derived organoids

**DOI:** 10.1093/jmcb/mjaa031

**Published:** 2020-07-11

**Authors:** Fong Cheng Pan, Todd Evans, Shuibing Chen

**Affiliations:** Department of Surgery, Weill Cornell Medical College, New York, NY 10065, USA

**Keywords:** human pluripotent stem cells, endoderm, gastrointestinal development, disease modeling

## Abstract

Recent advances in development of protocols for directed differentiation from human pluripotent stem cells (hPSCs) to defined lineages, in combination with 3D organoid technology, have facilitated the generation of various endoderm-derived organoids for *in vitro* modeling of human gastrointestinal development and associated diseases. In this review, we discuss current state-of-the-art strategies for generating hPSC-derived endodermal organoids including stomach, liver, pancreatic, small intestine, and colonic organoids. We also review the advantages of using this system to model various human diseases and evaluate the shortcomings of this technology. Finally, we emphasize how other technologies, such as genome editing and bioengineering, can be incorporated into the 3D hPSC-organoid models to generate even more robust and powerful platforms for understanding human organ development and disease modeling.

## Introduction

Over the last 10 years, improved techniques in organoid culture and handling have led to a dramatic surge in the number of studies using this model system to investigate human embryonic development and disease progression *in vitro*, complementing existing animal and 2D cell culture models. An organoid is a mini-organ formed *in vitro* in 3D culture from a single cell or group of cells that possesses the capability to self-organize, self-renew, and differentiate to different cell types associated with its corresponding organ, in response to appropriate signals. In the 1950s and 1960s, some studies referred to organoids as ‘intracellular organelles’, a term that is now obsolete (Duryee and Doherty, 1954). Others have used the word organoids to describe tumors (Godienko, 1964) or abnormal cellular growths (Wolter, 1967). Thus, the definition of organoids varies between fields of research depending on the origin of the organoids and methods of isolation (Shamir and Ewald, 2014; Simian and Bissell, 2016). Recent literature documents that organoids can be derived from any of several sources: (i) human pluripotent stem cells (hPSCs), including human embryonic stem cells (hESCs) and induced pluripotent stem cells (iPSCs), which can generate organoids representing a variety of organs in the body; (ii) adult organ-specific stem cells, which retain organ identity and have the ability to generate different cell types present in that organ; (iii) progenitor/normal cells of organs that lack stem cell populations such as the pancreas and liver; (iv) mature differentiated cell types of an organ; and (v) primary explants derived from normal tissues or tumors (Stange et al., 2013; Shamir and Ewald, 2014; Lancaster and Knoblich, 2014; Moore et al., 2015; Clevers 2016; Fatehullah et al., 2016; Drost and Clevers, 2017). Organoids representing different organs of the body have been developed for various applications including but not limited to the study of basic biological and developmental processes, disease modeling, and drug screening.

### A short history of the origin of organoids

While interest in organoids has increased dramatically over the past decade, in fact organoid-based research has been around much longer than most recent reviews imply. The first attempt to generate organoids was reported by Wilson (1910) in the early 1900s, in which he showed that single dissociated sponge cells were able to reaggregate and re-organize to form an entirely new viable sponge without any external cues. In subsequent decades, several laboratories used the same dissociation-and-reaggregation method to demonstrate that cells isolated from different organs of more complex organisms including Coelenterates (De Morgan and Drew, 1914), amphibians (Holtfreter, 1948), and chick (Moscona and Moscona, 1952) also have the ability to self-organize and re-establish the structural pattern of the original tissue while in suspension culture.

The emergence of modern organoid research started with the discovery and isolation of various extracellular matrix (ECM) components that enabled investigators to culture differentiated cells in 3D. Compared to 2D culture, 3D culture plays an important role in supporting cell growth, patterned cell differentiation, and development of more complex organ-like structures *in vitro*. Collagen was the first ECM molecule to be isolated and tested to affect cell viability; a layer of collagen was shown to improve the survival rate of cells cultured in 2D (Huzella, 1932; Ehrmann and Gey, 1956). Nevertheless, cells cultured under these conditions tend to lose differentiation capacity after a few days. Two decades later, it was shown that when primary hepatocytes (Michalopoulos and Pitot, 1975) or mammary epithelial cells (Emerman and Pitelka, 1977) were allowed to float in the media on collagen gels, these cell types maintained their differentiation status. Although the mechanism was not understood at the time, this suggested that eliminating contact with hard plastic while floating in a collagen gel provided a permissive environment for cell differentiation.

Between 1963 and 1977, three scientists isolated a gelatinous basement membrane while studying a chrondrosarcoma tumor that produced excessive ECM, and that was subsequently named after them as the Engrelbreth‒Horm‒Swarm (EHS) sarcoma. The ECM generated from the EHS sarcoma is now known as Matrigel, which is enriched in laminin, collagen IV, and fibronectin. With the discovery of these distinct ECM components, numerous groups independently demonstrated the importance of ECM in regulating gene expression in various primary cell types and therefore controlling the differentiation states and functions of these primary cells. In 1991, Streuli et al. (1991) showed that in mammary cells, the interaction of integrin with laminin-rich ECM is required for expression of the milk protein β-casein, providing the first mechanistic evidence that ECM regulates gene expression in orchestrating tissue function and morphogenesis.

With progressive understanding of the importance for interplay between growth factors, morphogens, and ECM in morphogenesis combined with advances in stem cell isolation and differentiation strategies, different methods have been devised for 3D culture of cells from different organs. First reported in 2008, Sasai and colleagues demonstrated self-organized and polarized cortical tissues derived from directed differentiation of either mouse or human ESCs using a 3D aggregation culture protocol called SFEBq (serum-free culture of embryoid body-like quick aggregates) (Eiraku et al., 2008). The following year, in a ground-breaking study, Sato et al. (2009) reported that when cultured in Matrigel, a single LGR5^+^ intestinal stem cell isolated from the adult mouse intestinal crypt is capable of forming a complex *in vivo-*like structure comprised of different intestinal cell types. In less than a decade, scientists have successfully generated organoids that mimic the brain, retina, kidney, liver, stomach, pancreas, intestine, and several other organs. For detailed information on the history of organoid development and use, readers can refer to a comprehensive review written by Simian and Bissell (2016).

### Pluripotent stem cells in disease modeling: 2D vs. 3D

The isolation of hESCs and generation of hiPSCs, collectively known as hPSCs, have fueled the interest for advancing cellular regenerative therapies. Over the past two decades, robust protocols have been described for directed differentiation of hESCs and hiPSCs into different cell types in 2D monolayer cell culture systems. However, a 2D culture system has several limitations. First, this system fails to accurately mimic the natural structure of tissues or tumors, and thus is incapable of precisely recapitulating complex morphogenetic processes underlying embryonic development, cellular differentiation, tissue regeneration, and tumor formation. Moreover, the patterning, polarity, and mode of cell division are also altered in 2D culture, resulting in changes in gene expression and cell function (Duval et al., 2017; Ho et al., 2018).

The search for a better model system has driven the development of 3D organoid culture for more accurate recapitulation of *in vivo* conditions. The organoid system has indeed been shown to be superior in several ways compared to 2D culture, because organoids have the ability to self-organize into remarkable tissue-like architecture and undergo multi-lineage differentiation containing heterogeneous populations of cells. The 3D organoid culture system also enables fundamental processes such as cellular migration and segregation, spatially restricted lineage commitment, and cellular organization to occur in a more physiologically relevant microenvironment. With the tractability and scalability in culturing methods, there has been significant progress in the generation of 3D organoids *in vitro* from hPSCs using knowledge gained over the years from many developmental biology studies in mouse and human.

After the directed differentiation of hPSCs into defined germ layers—ectoderm, mesoderm, or endoderm—progenitors of diverse lineages representing a specific germ layer can arise in 3D aggregates to allow further differentiation into different tissues of interest. Such methods have led to the generation of a myriad of organoid types that model different tissues/organs derived from hPSCs including small intestine (Spence et al., 2011), colon (Crespo et al., 2017; Munera et al., 2017), retina (Eiraku et al., 2008; Nakano et al., 2012; Kuwahara et al., 2015), brain (Lancaster et al., 2013), liver (Takebe et al., 2013), stomach (McCracken et al., 2014), lung (Dye et al., 2015), pancreas (Hohwieler et al., 2017), and kidney (Forbes et al., 2018). Disease modeling using hESC/hiPSCs-derived brain, retinal, lung, and kidney organoid cultures has been extensively reviewed elsewhere (Sasai, 2013; Lancaster and Knoblich, 2014; Barkaukas et al., 2017; Nishinakamura, 2019). In this review, we focus on the development of hPSC-derived endodermal lineage organoids including stomach, liver, pancreas, small intestine, and colon (Table 1). We discuss recent advances in the development of these organoids and highlight their promising potential in biomedical applications and translational precision medicine.

**Table 1:** Summary of hPSC-derived endodermal organoids used for modeling human gastrointestinal development and disease.

Tissue/organ	Organ development and disease models	References
Stomach	Antral stomach development; enteroendocrine specification; *H. pylori* infection	McCracken et al. (2014)
	Fundus stomach development	McCracken et al. (2017)
Liver (hepatocytes)	Steatohepatitis	Ouchi et al. (2019)
	HBV and HCV infection	Roelandt et al. (2012); Schwartz et al. (2012); Nie et al. (2018)
Liver (cholangiocytes)	Alagille syndrome	Ogawa et al. (2015); Sampaziotis et al. (2015)
	Cystic fibrosis	Ogawa et al. (2015); Sampaziotis et al. (2015)
Pancreas	Pancreatic cancer	Huang et al. (2015)
	Cystic fibrosis	Hohwieler et al. (2017)
	β cell functions/diabetes	Shim et al. (2015); Kim et al. (2016)
Small intestine	Regional patterning	Tamminen et al. (2015); Tsai et al. (2017)
	Enteroendocrine specification	Spence et al. (2011)
	Gastrointestinal virus infection	Finkbeiner et al. (2012)
	Gastrointestinal bacterial infection	Forbester et al. (2015); Leslie et al. (2015)
Colon	Colon development	Munera et al. (2017)
	Colorectal cancer	Crespo et al. (2017); Sommer et al. (2018)

## Stomach organoids

During development, the stomach epithelium forms invaginations into the lamina propria known as gastric units, which are subdivided into the pit and the gland (Wilson and Stevenson, 2019). The mature human stomach is divided into two distinct functional domains: the proximal fundus/corpus stomach and the distal pyloric antrum region.

**Figure 1:**
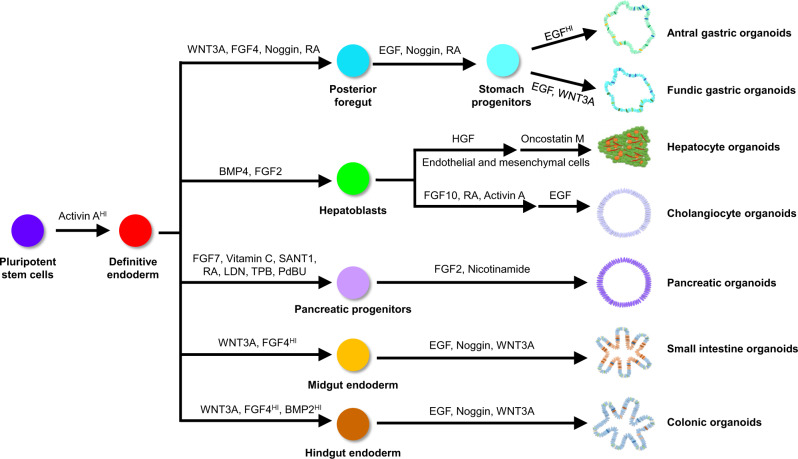
Schematic showing hPSC differentiation protocols toward distinct endodermal lineages, including gastric, hepatocyte, cholangiocyte, pancreatic, small intestine, and colonic organoids.

The knowledge obtained from studies of anterior endoderm patterning and stomach development in animal models including the mouse has been instrumental in generating the first human gastric organoids through directed differentiation of hESCs as pioneered by the Wells laboratory (McCracken et al., 2014; Table 1). In brief, the differentiation protocol involves differentiation of hESCs first toward definitive endoderm (DE) by treatment with Activin A. DE is then patterned to adopt a posterior foregut endoderm fate using a combination of WNT3A, FGF4, Noggin, and retinoic acid (RA). At this point, posterior foregut organoids that emerge from 2D culture are embedded in Matrigel and treated with RA, Noggin, and EGF for the following 72 h to induce stomach specification. The organoids are maintained in medium containing a high concentration of EGF for up to 34 days to promote morphogenesis and differentiation into various gastric cell types (Figure 1). With this protocol, McCracken et al. (2014) obtained gastric organoids containing cell types with antral stomach characteristics. Fundus glands were not specified, and eventual protocols toward that goal required knowledge of fundus stomach development.

Using hESC-derived antral gastric organoids, these investigators identified novel signaling mechanisms that regulate early endoderm patterning and gastric enteroendocrine cell differentiation upstream of NEUROG3, the master regulator of endocrine differentiation. Three years later, the same group reported the generation from hESCs of gastric organoids with fundus stomach characteristics (McCracken et al., 2017; Table 1) using a modified protocol specific for antral gastric organoids. In combination with *in vivo* mouse studies, they discovered that canonical Wnt signaling is required for fundus stomach specification. Thus, prolonged activation of Wnt signaling after the posterior foregut endoderm stage is able to produce fundus gastric organoids with different fundus-specific gastric cell types including chief cells, surface and neck mucous cells, and enteroendocrine cells (Figure 1). They also found that inhibition of MEK/ERK and activation of BMP signaling is required for differentiation of acid-secreting parietal cells in these gastric organoids. These gastric organoid systems can be used to study the physiological interactions of human gastric cells in normal and disease conditions, initiation and progression of gastric cancer by chronic *Helicobacter pylori* infection, and responses to pharmacological treatments.

## Liver organoids

### Establishment of a protocol for generation of hepatocyte organoids

Directed differentiation of hESCs and hiPSCs into hepatocyte-like cells was reported over a decade ago by several research groups (Baharvand et al., 2006; Agarwal et al., 2008; Si-Tayeb et al., 2010). A general hepatocyte differentiation protocol includes generation of DE from hPSCs by Activin A treatment, followed by specification of hepatoblasts using BMP4 and FGF2. The hepatoblasts are further differentiated into immature hepatocytes by HGF treatment and maturation of hepatocytes is achieved by culturing in medium containing Oncostatin M (Figure 1). Variations on this protocol have been reviewed elsewhere (Safinia and Minger, 2009). However, these differentiation protocols generate hepatocyte-like cells in 2D culture that fail to display important functional properties seen in complex tissues.

Early morphogenesis and specification of the formative liver bud depend highly on the interplay of signaling between hepatic endoderm cells with adjacent mesenchymal and endothelial progenitors (Zhao and Duncan, 2005). Based on this knowledge of developmental biology and in combination with an efficient hepatocyte-differentiation protocol established by Duncan and colleagues (Si-Tayeb et al., 2010), Takebe et al. (2013) generated the first vascularized hiPSC-derived liver organoids (more appropriately modeling liver buds) by co-culturing hiPSC-derived hepatic endodermal cells with human umbilical vein endothelial cells (HUVECs) and human mesenchymal stem cells (hMSCs). The contribution of hMSCs in this context is to differentiate into perivascular cells that stabilize the vasculature. Notably, although these cells were initially plated in 2D conditions, the co-culture system allowed the hiPSC-derived hepatocytes to self-organize into 3D clusters resembling liver buds. When transplanted *in vivo* into immune-deficient interleukin-2Rγ null (NSG) mice, the vasculature of hiPSC-derived liver bud organoids connected efficiently with host vessels forming a functional vasculature capable of inducing further functional maturation of hiPSC-derived hepatocytes. The hiPSC-derived liver organoids rescued and improved survival of the acute liver failure (ALF) mouse model. This co-culture method stresses the importance of multicellular interactions during liver development and provides a highly successful proof-of-principle approach likely applicable to other organ systems. Nonetheless, a major caveat of this study is that HUVECs and hMSCs are established cell lines that were not derived from the same hiPSCs, and without further modifications, this type of construct could trigger graft rejection upon transplantation.

To overcome this major issue, Nie et al. (2018)co-cultured hiPSC-derived hepatic endodermal cells, with endothelial cells and mesenchymal cells that were all derived from a single donor umbilical cord in a 3D-microwell platform to allow self-organization and formation of liver organoids. This approach generated liver organoids with enhanced hepatic capacity compared to using hiPSC-derived hepatocyte-like cells alone. Upon transplantation into the renal capsules of the ALF mouse model, the hiPSC-derived hepatocytes rapidly restored hepatic function and improved survival. While this represents an improved option as compared to the original strategy reported by Takebe et al. (2013), derivation of endothelial and mesenchymal cells from umbilical cord is time-consuming and requires optimized protocols.

An alternative approach reported by Wu et al. (2019) was to generate hepatobiliary organoids containing endothelial and mesenchymal cells within the same dish, excluding the need to separately differentiate endothelial and mesenchymal cells. This was achieved by retaining 25% of the pluripotency-supportive media, mTESR, in the differentiation protocol at the hepatic specification stage. The resulting hepatobiliary organoids generated with this protocol contain hepatocytes, cholangiocytes, and endothelial-like cells. Using this protocol, they further showed that the specification of biliary fate requires Notch signaling on cholangiocytes, which is activated by vascular endothelial growth factor (VEGF) from endothelial-like cells and transforming growth factor-β (TGF-β) present in mTESR media.

### Modeling fatty liver disease using hepatic organoids

**Figure 2:**
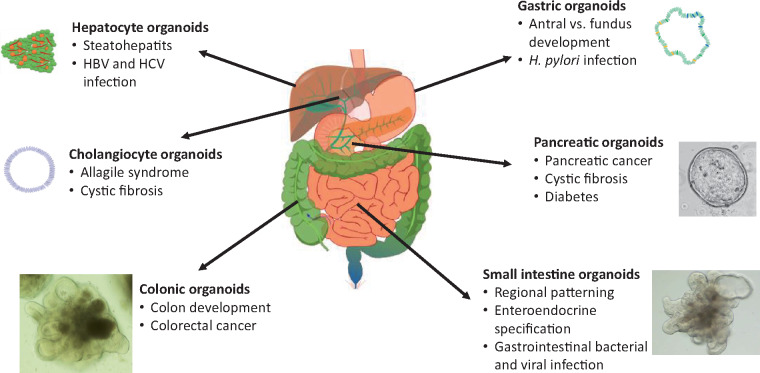
Schematic summarizing hPSC-derived endodermal organoids used for modeling human gastrointestinal development and disease.

Steatohepatitis is a type of fatty liver disease characterized by inflammation of the liver with concurrent fat accumulation (steatosis). Based on the successful generation of intestinal organoids containing both endodermal and mesodermal lineages (Spence et al., 2011; McCracken et al., 2017), Takebe’s group adopted these protocols for co-differentiating hepatocytes with other supporting stromal cell types, such as hepatic stellate cells and Kupffer cells in 3D cultures (Ouchi et al., 2019; Table 1). Upon treatment with free fatty acid, these liver organoids exhibited characteristic steatosis and elicited an inflammatory response leading to the formation of fibrotic organoids, in which the organoid stiffness was measured by atomic force microscopy. This new platform was then used to model Wolman disease, a pathology caused by the defective activity of lysosomal acid lipase (LAL). Hepatocytes from patients with Wolman disease have massive lipid accumulation accompanied by extensive fibrosis and lethal steatohepatitis. Wolman hiPSC-derived liver organoids also showed a significant increase in lipid accumulation, which could be rescued by treatment with recombinant LAL. This study further demonstrated a clinically translational aspect for this model by showing that obeticholic acid, a farnesoid X receptor agonist, is able to stimulate ileal expression of FGF19. FGF19 in turn exerts direct effects on the liver to suppress lipid accumulation, thus alleviating steatohepatitis in hiPSC-derived liver organoids from Wolman patients (Figure 2). This platform could be used to model other chronic liver diseases such as inborn error of hepatic metabolism and alpha-1-antitrypsin (A1AT) diseases.

### Liver organoid model of chronic viral infection pathogenesis

The liver is the target organ of many viruses including hepatitis B (HBV) and C (HCV). Evidence suggests that host genetic background might explain the heterogeneous outcome of these infections (Burgner et al., 2006). Since research on host‒pathogen interactions using the established hepatic cancer cell lines or humanized mouse models does not represent individual host genetic backgrounds, this limits their application for understanding the potential for host genetics to impact viral pathogenesis. HCV infection models using hESC- and hiPSC-derived 2D hepatocyte cultures (Roelandt et al., 2012; Schwartz et al., 2012; Table 1) and HBV infection models using 3D liver organoids (Nie et al., 2018) have been reported. These studies demonstrated that PSC-derived hepatocytes are able to support the entire life cycle of these viruses, including recapitulation of natural entry of HBV and HCV, cell‒cell transmission, and capacity for eliciting an inflammatory response of hepatocyte to infection (Figure 2). Nie et al. (2018) further showed that the PSC-derived liver organoids are more susceptible to HBV infection and better model subsequent hepatic dysfunction, compared to hPSC-hepatocyte-like cultured in 2D conditions. Whether such models can be used for scaled virus production for research purposes and for screening of patient-specific antiviral drugs awaits further investigation.

### Cholangiocyte organoids for modeling Alagille syndrome and cystic fibrosis

Cholangiocytes are epithelial cells that form the adult biliary ductal system that regulates bile homeostasis and modulate inflammatory responses, as well as participates in repair of liver injuries. Several protocols have been reported for differentiating cholangiocytes from hPSCs; each used similar initial differentiation steps in order to generate hepatoblasts, and then used variations in order to specify and mature cholangiocytes (Dianat et al., 2014; Ogawa et al., 2015; Sampaziotis et al., 2015, 2017). Dianat et al. (2014) reported the use of growth hormone, EGF, interleukin-6, and sodium taurocholate with culture on collagen I for cholangiocyte differentiation and maturation. This protocol produced a sub-population of cholangiocytes known as small cholangiocytes, similar to those normally located in the canal of Hering (Sampaziotis et al., 2017). By co-culturing hepatoblasts with mouse OP-9 bone marrow stromal cells in the presence of EGF, HGF, and TGF-β and in ECM comprised of collagen type-1 and Matrigel, Ogawa et al. (2015) were able to generate mature cholangiocyte organoids with characteristics of large cholangiocytes. The OP-9 stromal cells likely support the specification and maturation of cholangiocyte organoids by providing niche factors such as JAG1, which activates Notch signaling necessary for cholangiocyte development. This method, however, has significant limitations for mechanistic studies in biliary development, since additional unknown factors may be secreted by OP-9 cells. To overcome this caveat, Vallier’s group developed a different strategy, in which specification of cholangiocyte progenitors from hepatoblasts was induced by FGF10, RA, and Activin A, followed by induction of cholangiocyte maturation in 3D using Matrigel and medium containing EGF (Sampaziotis et al., 2015, 2017; Figure 1). Using this more defined method, they were able to generate cholangiocyte organoids with functional characteristics of large cholangiocytes similar to those generated by Ogawa et al. (2015).

With successful generation of functionally mature cholangiocyte organoids, these platforms were used to model the pediatric biliary disorder Alagille syndrome, which is characterized by bile duct paucity caused by mutations in JAG1 or NOTCH2 (Ogawa et al., 2015; Sampaziotis et al., 2015; Table 1; Figure 2). By blocking Notch signaling in cholangiocyte organoids using γ-secretase inhibitors, organoid formation and ductal branching morphogenesis of the cholangiocyte organoids were inhibited. These findings reinforce previous observations in mouse models and demonstrate the importance of Notch signaling in human cholangiocyte specification.

Cystic fibrosis is an autosomal recessive disorder caused by mutation of the *cystic fibrosis transmembrane conductance regulator* (*CFTR)* gene, which encodes a sodium chloride transporter. Loss or dysfunction of CFTR protein in cholangiocytes results in reduced intraluminal chloride secretion, increased bile viscosity, and biliary obstruction causing biliary fibrosis and cirrhosis (Colombo, 2007; Staufer et al., 2014). hiPSC-derived cholangiocyte organoids were used to model polycystic and cystic fibrosis-associated cholangiopathies (Ogawa et al., 2015; Sampaziotis et al., 2015; Figure 2; Table 1). CF-hiPSC-derived cholangiocyte organoids recapitulated major phenotypes of cystic fibrosis-related cholangiopathies and were used to identify new patient-specific therapeutic agents that could potentially alleviate the cholangiopathy-related symptoms in these patients.

## Pancreatic organoids for modeling of pancreatic cancer, cystic fibrosis, and diabetes

The pancreas is derived from posterior foregut endoderm and consists of endocrine and exocrine compartments. The endocrine compartment is comprised of five major hormone-secreting cell types and involved in regulating glucose homeostasis. The two major cell types in the exocrine compartment are the acinar cells that produce digestive enzymes and the ductal cells that secrete mucus and bicarbonate solution for delivering digestive enzymes to the intestine (Pan and Wright, 2011).

Various established pancreatic differentiation protocols have been reported (Rezania et al., 2014; Pagliuca et al., 2014; Russ et al., 2015; Ghazizadeh et al., 2017; Amin et al., 2018) and extensively reviewed elsewhere (Hohwieler et al., 2019). These protocols have mostly focused on making pancreatic β cells in 2D culture for modeling diabetes (Zeng et al., 2016; Guo et al., 2017; Zhou et al., 2018; Figure 2). Each of the protocols involves the allocation of DE into a posterior gut tube fate using FGF7. Stages 2 and 3 of differentiation involve specification of posterior gut tube to posterior foregut endoderm and subsequently pancreatic progenitors induced with several signaling molecules including FGF7, RA, vitamin C, and a protein kinase C agonist, while inhibiting BMP and Shh signaling. Adapting similar differentiation protocols to generate pancreatic progenitors, pancreatic organoids have also been generated from hPSC for modeling pancreatic cancer and cystic fibrosis (Huang et al., 2015; Hohwieler et al., 2017; Table 1).

Huang et al. (2015) used a two-step protocol for differentiation of hPSC-derived pancreatic progenitors toward exocrine fate by inhibiting TGF-β and Notch signaling and obtained pancreatic organoids expressing low levels of ductal and acinar markers. These pancreatic organoids underwent further morphogenesis and differentiation into mature ducts and acini upon *in vivo* transplantation into the mouse mammary fat pad. This organoid platform was further used to model initiation and formation of pancreatic ductal adenocarcinoma (PDAC) (Figure 2). The hallmark of PDAC progression is an activating mutation of KRAS in combination with the loss of multiple tumor suppressors such as TP53, CDKN2A, and SMAD4. Huang et al. (2015) generated pancreatic organoids carrying either a single KRAS-activating mutation or a p53^R172H^ dominant-negative mutation. These organoids formed cystic structures with morphology similar to pancreatic intraepithelial neoplasia (PanIN) lesions both *in vitro* and *in vivo*. It is noteworthy that these PanIN organoids were not able to expand to form larger PanIN lesions or progress to the PDAC tumor stage upon *in vivo* transplantation into the mouse fat pad. It remains unknown how these organoids would behave if orthotopically transplanted in their natural environment. An interesting approach would be to generate hPSC-derived pancreatic organoids containing combinations of different driver mutations to model PDAC progression and metastasis.

The pancreas is one of the organs most seriously affected by cystic fibrosis. The CFTR protein is highly expressed in pancreatic ductal epithelial cells that are associated directly or indirectly with bicarbonate transport. In the pancreas of cystic fibrosis patients, defective bicarbonate secretion leads to increased mucus viscosity, hyperconcentration of digestive enzymes, and subsequently obstruction of ductal epithelium, which can lead to pancreatitis, cystic fibrosis-related diabetes mellitus, and ultimately destruction of the whole organ (Cutting, 2015). Hohwieler et al. (2017) adapted a differentiation protocol, in which pancreatic organoids with mature ductal and acinar cells were generated *in vitro* without a need for *in vivo* maturation (Figure 1). With this protocol, pancreatic organoids were generated using hiPSCs from patients with cystic fibrosis and used to identify compounds that can improve cellular processing and gating function of the CFTR protein. Furthermore, this platform facilitated testing of a new strategy for screening a panel of chemically modified mRNAs (cmRNAs) for gene complementation by transfection of the CF-hiPSC-pancreatic organoids to identify cmRNA that can complement *CFTR* gene function. This strategy could, in the future, be applied to other organoids being used to model cystic fibrosis including cholangiocyte, lung, and colon organoids.

As mentioned above, most studies using hPSC-derived β cell differentiation have been used to model diabetes using 2D cultures. Two studies have demonstrated that aggregating hESC-derived differentiated hormonal cell types to form islet-like structures leads to enhanced insulin secretion, indicating that aggregation improves β cell function (Shim et al., 2015; Kim et al., 2016; Table 1). It is worth mentioning that a recent study showed that transcription factor-based reprogramming of ductal cells to β cells *in vitro* was significantly improved when mouse ductal organoids were used compared to 2D human ductal cell lines (Azzarelli et al., 2019). Whether such efficient reprogramming can be achieved in hPSC-derived pancreatic organoids awaits future investigation.

## Small intestinal and colon organoids

The mature intestine is a highly compartmentalized organ with distinct regions that fulfill different functions including digestion, absorption, metabolism, and immunity. The small intestine, which plays major roles in digestion and nutrient absorption, consists of the proximal duodenum, jejunum, and distal ileum. The large intestine, which is responsible for feces formation as well as absorption of water, electrolytes, and vitamins produced by gut bacteria, is comprised of cecum, colon, rectum, and anus. Dysregulation of intestinal development or function, as well as pathogen infection, can cause a range of intestinal-associated diseases such as inflammatory bowel disease, short bowel syndrome, infection-associated enteritis, and colorectal cancer. Before the development of intestinal organoids, these intestinal diseases were modelled using cell lines and explanted tissues, which have significant limitations including the lack of complex 3D architecture and cellular heterogeneity.

In 2011, the Wells group generated the first hPSC-derived intestinal organoids, based on the knowledge of developmental biology obtained from various model organisms including *Xenopus*, zebrafish, and mouse (Spence et al., 2011; McCracken et al., 2011). During development, the small intestine and large intestine are derived from DE that is specified into midgut and hindgut endoderm (Zorn and Wells, 2007). Therefore, to generate intestinal organoids, hPSCs were first differentiated into DE by treatment with Activin A, followed by patterning of DE into CDX2^+^ midgut and hindgut using a high concentration of WNT3A and FGF4 for 4 days. During this patterning step, monolayer epithelium undergoes morphogenesis and folding to generate 3D spheroids that ultimately bud off from the 2D epithelium. These 3D spheroids were further cultured in Matrigel using a minimal pro-intestinal differentiation medium containing niche factors including EGF, Noggin, and WNT3A (Figure 1). This protocol generated organoids with complex 3D architecture containing both epithelial and mesenchymal components of the small intestine. The epithelium component of the organoids consists of enterocytes that form villus-like structures, crypt-located LGR5^+^ transit-amplifying (TA) and stem cells, enteroendocrine cells, Paneth cells, and goblet cells. Spence et al. (2011) showed the feasibility of using this intestinal organoid platform for developmental studies by knocking out or overexpressing NEUROG3, a master regulator of endocrine fate (Table 1). Nevertheless, the regional identity of these intestinal organoids remained elusive. A follow-up study by Tsai et al. (2017) demonstrated that the regional specification of these small intestinal organoids can be achieved by exposing DE to high concentrations of WNT3A and FGF4 for different lengths of time. Shorter exposure time led to duodenum fate, while longer exposure time generated organoids with characteristics of ileum. However, long-term exposure of mid/hindgut progenitors to FGF4 inhibits differentiation and maturation of intestinal organoids (Tamminen et al., 2015; Table 1). While WNT3A and FGF4 are required for small intestine specification, transient activation of BMP2 signaling is sufficient to drive the hPSC-derived gut tube into colonic fate, as high levels of BMP signaling are required for posterior gut tube and large intestine development (Munera et al., 2017; Figure 1). Taken together, these studies showed that intestinal organoid differentiation *in vitro* can largely recapitulate *in vivo* human intestinal development, allowing this system to be used for studying genetic diseases related to human intestinal development (Figure 2).

Since the initial publication of the hPSC-derived intestinal organoid differentiation protocol, various modifications have been reported. In one strategy, Forster et al. (2014) transplanted into mice hESCs containing an LGR5-GFP reporter gene to generate teratomas that contain LGR5^+^ putative intestinal cells, which were isolated and cultured in 3D to form intestinal organoids. Intestinal organoids generated using this method contain mature intestinal cell types, including LGR5^+^ adult intestinal stem cells. However, the frequency and efficiency of generating teratoma containing LGR5^+^ intestinal cells are unknown, which limits practical application. Takahashi et al. (2018) reported that the addition of WNT3A and FGF2 together with Activin A enhanced the efficiency of DE differentiation, which subsequently increased the frequency of spheroid formation during the mid/hindgut patterning stage. In the same study, a Matrigel-free suspension-based protocol was developed for culturing intestinal organoids using a commercially available medium called Happy Cell ASM 3D Culture Medium. The use of such suspension organoid cultures without requiring a scaffold could facilitate recovery of the organoids for various assays, particularly drug screening, and is important for future translation of clinical applications.

In another study, enterospheres containing anterior NKX2.1^+^ lung progenitors and posterior CDX2^+^ mid/hindgut intestinal progenitors were derived from DE generated using a previously established 11-day monolayer differentiation protocol (Nadkarni et al., 2017). The lung progenitors were progressively depleted over time and the majority of cells in the enterosphere became CDX2^+^ intestinal cells when cultured in 3D Matrigel and MTEC medium, a type of medium suitable for growing most cell types. This study also emphasized the importance of different growth factors in the culture media for inducing differentiation toward diverse intestinal cell types or maintenance of stem cell populations in the enterosphere. While a combination of WNT3A, EGF, Noggin, and R-spondin is sufficient for differentiation of enterocytes, enteroendocrine and Paneth cells, inhibition of Notch signaling dramatically induced goblet cell specification. Furthermore, the LGR5^+^ stem cell population is significantly enriched by augmenting the medium with gastrin and nicotinamide, together with inhibition of TGF-β and p38 MAPK signaling, allowing long-term propagation of the enterospheres (Nadkarni et al., 2017).

Most studies showed that the maturation of hPSC-derived intestinal organoids can only be achieved by *in vivo* transplantation underneath the kidney capsule of NSG mice (Watson et al., 2014; Finkbeiner et al., 2015; Munera et al., 2017; Tsai et al., 2017). Using the *in vivo* transplantation strategy, the maturation process of human intestinal organoids during the fetal-to-adult transition has been elucidated (Finkbeiner et al., 2015; Table 1). Furthermore, such methods can be used to identify systemic humoral signals during injury or regeneration that promote the maturation and expansion of intestinal cell types (Watson et al., 2014). One noteworthy observation is that the mesenchymal compartment of the intestinal organoids plays an important role in the expansion and engraftment of these organoids, as organoids without mesenchyme failed to expand *in vitro* or engraft *in vivo* (Watson et al., 2014; Nadkarni et al., 2017).

### Modeling colorectal cancer using intestinal organoids

Colorectal cancer is the second leading cause of cancer death worldwide. Mutation of the *adenomatous polypopsis coli* (*APC*) gene is the main driver of colorectal cancer, found in ∼85% of cases. Recent advances in gene-editing technology coupled with protocols for hPSC-derived intestinal organoids have allowed modeling of colorectal cancer with individual driver mutations, with a particular focus on the initiation events of this disease. Crespo et al. (2017) generated colon organoids using hiPSC derived from familial adenomatous polypopsis (FAP) patients carrying germline mutations of the *APC* gene, in order to model FAP pathogenesis *in vitro* (Table 1). The FAP-hiPSC colon organoids exhibited enhanced Wnt signaling activity and showed increased epithelial proliferation. This platform was then used to screen for drugs that could inhibit Wnt activity and restore normal proliferation of the FAP organoids. In another study, isogenic iPSC-derived intestinal organoids containing APC mutations were used to show that APC heterozygosity in these organoids is sufficient to induce changes in cell polarity and identity, gene expression patterns, as well as chromosomal aberrations (a hallmark of colorectal cancer), providing novel insights into the earliest initiation events of APC-mediated colorectal cancer tumorigenesis (Sommer et al., 2018; Figure 2; Table 1). In the future, use of this hPSC-derived colon organoid platform will contribute to understanding how other major mutations contribute to colorectal cancer progression and metastasis.

### hPSC-intestinal organoids for modeling gastrointestinal bacterial and viral infections

Gastrointestinal infections from bacteria, viruses, and parasites cause gastroenteritis leading to symptoms such as diarrhea, vomiting, and abdominal pain. The ability to fully understand how these pathogens interact with human intestinal cells in order to cause disease has been hampered by the inability to efficiently isolate, culture, and propagate the pathogens *in vitro*, particularly for enteric viruses. Human intestinal organoids, derived from either hPSCs or adult stem cells, have recently been used to study the pathogenesis of some of the infectious pathogens that had been difficult to grow in 2D cell lines. The first report using hPSC-derived intestinal organoids to model viral gastrointestinal infections came from the Estes group (Finkbeiner et al., 2012; Table 1), who showed that these organoids were capable of supporting the replication of clinically isolated rotaviruses and production of infectious virus progeny. Surprisingly, this virus is capable of infecting both epithelial and mesenchymal components of the intestinal organoids, indicating that this is a promising platform not only to model rotavirus infection but other viral-related causes of gastroenteritis (Figure 2).

Enteric bacterial-related gastrointestinal infection, especially those caused by pathogenic *Salmonella species* are responsible for >90 million cases of gastroenteritis and 20 million cases of typhoid per year (Mather, 2013). Mouse models are commonly used to study the pathogenesis of *Salmonella*-related gastroenteritis but the disease and symptoms differ in mouse and human, prompting the need for a better model of this infection (Mather, 2013). By injecting *Salmonella enterica serovar Typhimurium* into the lumen of hPSC-intestinal organoids, Forbester et al. (2015) showed that in the infected organoids the bacteria were able to invade the intestinal epithelium, form intracellular *Salmonella*-containing vacuoles, and induce cytokine gene expression (Table 1). While it is relatively simple to cultivate and study pathogenic mechanisms of aerobic bacteria *in vitro*, growing anaerobic bacteria in cell lines has been a challenge, since growing intestinal cells require oxygen. Leslie et al. (2015) showed that *Clostridium difficle*, an anaerobe and common cause of infectious nocosomial diarrhea, can survive up to 12 h when injected into the lumen of the hPSC-derived intestinal organoids, allowing the interaction of the bacteria with host epithelium to be studied. Upon infection, this bacterium caused disruption of intestinal epithelium, resulting in loss of paracellular barrier function, which is mainly mediated by toxin TcdA. These studies highlight the promise of this platform for understanding disease mechanisms causing other bacterial and protozoan-related gastroenteritis, both aerobic and anaerobic, which have not been previously investigated in other models. This platform could also be used to search for better treatments via drug screening to target pathogens, for which there are no efficient therapeutic agents.

## Advantages of hPSC-derived organoids to study human diseases

We have highlighted some of the advantages of using the endodermal organoid platform for modeling development and disease. Here, we briefly summarize the overall advantages of this system. Clearly, hPSC-derived endodermal organoids offer several significant advantages over 2D culture systems in addressing various aspects of human gut developmental biology, including lineage fate specification and regional organ patterning. Since the hPSC-to-organoid differentiation process resembles to a large extent *in vivo* development, hPSC-derived organoids provide a unique model to study mechanisms underlying embryonic defects and diseases that are associated with these defects. Together with advances in gene-editing technology, targeted mutations or correction of mutated genes can be performed relatively efficiently at the hPSC level before differentiating into different lineages and at the organoid level prior to autologous transplantation.

Furthermore, a major advantage of this system is the unlimited supply of the starting materials from which these organoids can be generated due to the ability of hPSCs to self-renew and differentiate. This subsequently allows large-scale organoid production for various assays, including drug screening and *in vivo* transplantation. Drug screening and validation of drug derivatives in animal models is not only costly but also carries uncertainty of efficacy due to fundamental differences in physiology between human and mouse models. The hPSC-derived organoids thus offer a cost-efficient and reliable approach to overcome these limitations.

## Limitation of hPSC-derived organoids

The hPSC-organoid system is not without its limitations. While some of the described hPSC-organoid models co-develop the supporting mesenchyme during the differentiation process, the system still generally lacks several stromal components such as the vasculature, neurons, and lymphatic and immune cells that preclude it from modeling diseases in which these stromal components play an important role in disease development. In addition, *in vivo* engraftment of organoids is highly enhanced by the presence of mesenchymal cells (Watson et al., 2014; Nadkarni et al., 2017) or co-culturing of organoids with endothelial cells prior to transplantation (Takebe et al., 2013). Thus, it seems highly likely that optimization of methodologies for co-culturing of organoids with different stromal components will be required for faithful modeling of the disease microenvironment, for example as shown by recently reported air‒liquid interface cultures for tumor organoids (Li et al., 2016; Neal et al., 2018).

Most studies rely upon *in vivo* engraftment to achieve maturation of organoids (Finkbeiner et al., 2015; Huang et al., 2015; Nadkarni et al., 2017). Based on the idea that adult intestinal epithelium interacts with immune cells in the intestinal mucosa to maintain intestinal homeostasis and mucosal immunity, and that the immune cells might play an important role in maturation of intestinal cells, recent attempts were made to generate mature hPSC-derived intestinal organoids *in vitro* by co-culturing with Jurkat T cells (Jung et al., 2018). Interleukin-2 secreted from Jurkat T cells activated STAT3 signaling and was responsible for promoting maturation of the hPSC-derived intestinal organoids. It would be interesting to know whether similar principles could be applied to promote the maturation of other hPSC-derived endodermal organoids.

Matrigel and type I collagen are two commonly used ECM components used to support the growth of 3D organoid cultures *in vitro*. There are several drawbacks associated with using these ECM components. First, they are produced by animal cell lines, which precludes their use for any application in human clinical trial studies. In addition, there is batch-to-batch variation in growth factors and ECM concentration during manufacturing, particularly in the production of Matrigel. Although growth factor-reduced versions of Matrigel are available, the ill-defined ECM concentrations could also affect the stiffness and porosity of the polymerized Matrigel. This could lead to lack of consistency in organoid differentiation, since stem and progenitor cell formation and differentiation of various cell types require different stiffness of ECM support (Gjorevski et al., 2016). Furthermore, methods for recovering organoids from the ECM scaffolds for downstream processing are relatively complicated and might affect the downstream analysis (Takahashi et al., 2018). Thus, alternative methods for culturing human organoids are required. Long-term culturing and clinical application will warrant a transition to a more defined and mechanically dynamic ECM to fully exploit the potential of hPSC-organoid technology. An animal-free hybrid PEG hydrogel scaffold for culturing intestinal organoids was reported by Gjorevski et al. (2016) and Cruz-Acuna et al. (2017), in which the matrix stiffness can be dynamically regulated. Such designer matrices with highly customizable scaffold structures could be the next-generation matrices that expand the applicability of organoids in human research studies.

## Perspectives

### Bioengineering technologies meet hPSC-organoid technology

The possibility of generating human organoid cultures that resemble specific endoderm-derived organs has opened up enormous possibilities for using organoids not only for disease modeling but as a source for cell replacement therapies or even whole organ transplantation. In the next 5‒10 years, we envision much more insight into the influence of various stromal cell types on the expansion and maturation of hPSC-derived organoids. Culturing of different stromal cell types often requires ECM composition and stiffness that are different for optimum organoid growth. Thus, finding an optimal condition for co-culturing these cell types with organoids will be challenging. More studies focusing on integrating bioengineering technology with hPSC-derived organoids might promote the discovery of such ideal co-culturing conditions. This will require the development of improved synthetic ECM, in which the mechanical features such as stiffness can be spatially and temporally modulated. A few of these regulatable synthetic ECMs have been used to study cell migration (Raeber et al., 2005; Kloxin et al., 2009; Guvendiren and Burdick, 2012). This includes photodegradable hydrogels containing photolabile (Kloxin et al., 2009) or photoinitiator (Guvendiren and Burdick, 2012) moieties within the network backbone of the hydrogel, allowing spatial and temporal softening or stiffening of the ECM using different light wavelengths. In addition to modulation by light, fine-tuning of ECM stiffness can also be achieved with pH adjustment by incorporating a combination of pH-sensitive poly(2-(diisopropylamino) ethyl methacrylate) (PDPA) and biocompatible poly(2-(methacryloyloxy) ethyl phosphorylcholine) (PMPC) components into the hydrogel backbones. Variation and application of these synthetic scaffolds have been extensively reviewed by Silva et al. (2019). In the future, these regulated synthetic scaffolds will not only allow optimum co-culturing of different cell types with organoids, but can also be used to establish growth factor gradients in the culture, both spatially and temporally, allowing differentiation and functional states to be precisely controlled.

The ability to generate a native cell-free tissue scaffold that could support partial or even whole organ growth *in vitro* would dramatically move the field toward whole organ transplantation. Kitano et al. (2017) showed that a decellularized rat intestine matrix, consisting of only the ECM of the intestine, can be repopulated with hPSC-derived intestinal organoids, which then differentiate into a monolayer of polarized intestinal epithelium. However, when the scaffold was repopulated with both human endothelial cells and hPSC-derived organoids, intestinal architecture with crypts- and villus-like structures was enhanced with an extensive functional vasculature. This strategy was successfully applied to a decellularized porcine intestine matrix (Kitano et al., 2017). With the possibility of including other intestinal supporting cell types, such implantable bioengineered intestine grafts generated from patient-derived organoids would provide an alternative treatment strategy for patients suffering from short bowel syndrome or even colorectal cancer.

### Faithful disease modeling in vivo using humanized mouse models

Another area needing attention is the development of an improved *in vivo* platform for precise engraftment that can faithfully model organ development as well as disease pathogenesis and progression. Currently, most maturation and assessment of hPSC-derived organoid function and mutant phenotype are done by *in vivo* transplantation into immune-deficient NSG mice, which lack a competent immune environment. This limits the use of these models to explore the interaction of epithelial cells with the immune system in certain inflammatory diseases. The recent development of humanized mouse models can overcome this limitation allowing the pathology of these diseases to be modelled more accurately. A humanized mouse model is an immune-deficient mouse engrafted with human hematopoietic stem cells that can give rise to a variety of human immune cells throughout the life of the animal (Lan et al., 2006; Kalscheuer et al., 2012; Lavender et al., 2013). Due to the presence of an intact human immune system, such humanized mouse models are particularly valuable for modeling disease pathology, as a powerful preclinical model for evaluation of novel therapeutics in an *in vivo* setting without putting patients at risk. In the future, the tandem use of this platform with hPSC-derived organoid technology will provide a more rigorous and holistic approach for modeling numerous types of complex human diseases such as inflammatory bowel disease, infectious diseases, and various cancers.

### Genome editing and hPSC-derived organoids

Advances in genome-editing technologies such as CRISPR-Cas9 open up possibilities for performing gene correction or studying specific mutations in development and disease. Gene editing in hPSC-derived organoids can already be applied to determine whether patient-specific mutations are pathogenic and to test potential novel therapies for precision medicine. Many challenges remain before gene-edited hPSC-derived organoids can be engrafted into humans for treating diseases; these include the relatively low efficiency of single-base gene editing and off-target effects caused by Cas9. With the establishment of efficient gene editing in organoids, editing in hPSC-derived organoids now can be performed at either of two stages, the pluripotent stem cell stage or the organoid stage, which allows stage-specific gene functions to be studied at both levels. In the near future, these technologies should be leveraged to generate inducible gene knockout or overexpression models that allow dissection of spatiotemporal impacts caused by specific mutations, particularly for genes that have multiphase functions. In addition, the development of additional cell type-specific reporters will facilitate monitoring the differentiation efficiency, for *in vivo* lineage-tracing upon engraftment or for isolation of specific cell types after *in vivo* transplantation for downstream analysis.

**Conflict of interest:** none declared.
